# Does *Gender* Leave an Epigenetic Imprint on the Brain?

**DOI:** 10.3389/fnins.2019.00173

**Published:** 2019-02-27

**Authors:** Laura R. Cortes, Carla D. Cisternas, Nancy G. Forger

**Affiliations:** Neuroscience Institute, Georgia State University, Atlanta, GA, United States

**Keywords:** sex, gender, epigenetics, stress, cosmetics, alcohol

## Abstract

The words “sex” and “gender” are often used interchangeably in common usage. In fact, the Merriam-Webster dictionary offers “sex” as the definition of gender. The authors of this review are neuroscientists, and the words “sex” and “gender” mean very different things to us: sex is based on biological factors such as sex chromosomes and gonads, whereas gender has a social component and involves differential expectations or treatment by conspecifics, based on an individual’s perceived sex. While we are accustomed to thinking about “sex” and differences between males and females in epigenetic marks in the brain, we are much less used to thinking about the biological implications of gender. Nonetheless, careful consideration of the field of epigenetics leads us to conclude that gender must also leave an epigenetic imprint on the brain. Indeed, it would be strange if this were not the case, because all environmental influences of any import can epigenetically change the brain. In the following pages, we explain why there is now sufficient evidence to suggest that an epigenetic imprint for gender is a logical conclusion. We define our terms for sex, gender, and epigenetics, and describe research demonstrating sex differences in epigenetic mechanisms in the brain which, to date, is mainly based on work in non-human animals. We then give several examples of how gender, rather than sex, may cause the brain epigenome to differ in males and females, and finally consider the myriad of ways that sex and gender interact to shape gene expression in the brain.

## Sex and Gender

Most animals on earth come in two sexes. From a biological perspective, sex is defined by gamete size within a species: animals with large gametes (i.e., eggs) are female and those with small gametes (i.e., sperm) are male (Maynard Smith, 1978). In mammals, eggs are made in ovaries and sperm in testes, so gonad type is often used as a shorthand for defining sex. Intersex gonads (part testis-part ovary) are very rare, so biological sex in mammals is a largely dichotomous variable.

Which gonad develops is determined by chromosomal sex (XX versus XY). If a Y chromosome is present, a gene cascade is initiated that causes the previously undifferentiated gonads to become testes; in the absence of a Y chromosome, an alternate cascade leads to the differentiation of ovaries (Brennan and Capel, 2004; Bowles and Koopman, 2013). The testes produce an androgenic steroid hormone, testosterone, for a brief perinatal period, and this hormonal exposure is responsible for masculinization of the external genitalia, internal duct systems, and other somatic differences (Jost, 1978). Testosterone also enters the developing brain and acts via androgen receptors or, after aromatization to an estrogen, via estrogen receptors to cause many of the known neural sex differences in animals (Morris et al., 2004; Forger et al., 2016; McCarthy et al., 2017).

Thus, biologists define “sex” based on what gonad is present and, in most cases, the chromosomal, gonadal, hormonal, and anatomical sex are all in accord. In individuals with Differences of Sexual Development, however, this is not the case, e.g., chromosomal males who have testes, but do not make the receptors to respond to testosterone, or chromosomal females exposed to excess androgens early in development (Lee et al., 2016).

In contrast to the relatively well-accepted delineation of sex, suggested definitions of “gender” are more varied. The Canadian Institutes of Health Research defines gender as, “*socially constructed roles, behaviors, expressions and identities of girls, women, boys, men, and gender-diverse people. It influences how people perceive themselves and each other, how they act and interact, and the distribution of power and resources in society*” (CIHR, 2015). Most of the work on epigenetics in the brain has been performed on experimental animals, which complicates the job of this essay because it is debatable whether non-human animals have gender, based on this definition. If gender requires socially constructed norms, and that an individual identifies as one sex or the other, it is hard to demonstrate gender in non-human animals. On the other hand, to the extent that gender is based on how you are treated by conspecifics, or to the “power and resources” you are likely to accrue, there are many examples of gender in the animal world. The biologist Joan Roughgarden has suggested defining gender simply as, “*the appearance, behavior, and life history of a sexed body*” (Roughgarden, 2009). Most social scientists embrace a definition of gender as a “*system that restricts and encourages patterned behavior*” (Risman and Davis, 2013). In other words, the emphasis is not on the individual (i.e., gender identity) but on social interactions that steer the individual’s behavior in different ways, based on their biological sex.

Given the latter two definitions, it may be argued that animals have gender, and this is how we define gender for the purposes of this review. Biological sex and gender often interact in complicated ways. However, we will refer to something as a “sex difference” when the difference appears to be due to factors such as sex chromosomes or gonadal hormones, and as a “gender difference” when the difference is likely due to social factors, i.e., when an individual is treated differently by conspecifics due to the individual’s perceived sex.

## Epigenetics

Epigenetic modifications determine what genes are expressed and represent mechanisms by which the genome can respond to environmental stimuli. The word “epigenetic” (literally, above genetics) was coined by C.H. Waddington in the 1950s to explain how different phenotypes can emerge from the same genotype. In other words, individuals (or cells) with the same genes may wind up with very different observable characteristics (phenotypes) based on environmental interventions at key developmental stages (Waddington, 1957). What controlled those changes was mysterious at the time, but many of the molecular mechanisms underlying the phenomena envisioned by Waddington have now been identified.

The DNA in every cell nucleus is packaged into chromatin by winding around histone proteins. The two best-understood types of epigenetic modifications are (1) post-translational modifications to histones, such as acetylation or methylation, and (2) covalent modifications to the DNA strand itself, e.g., by the addition of methyl or hydroxymethyl groups (Stricker et al., 2017). These epigenetic modifications are controlled by enzymes (e.g., histone acetyltransferases or DNA methyltransferases) and, once placed, they influence the likelihood that a given gene is expressed. For example, DNA methylation is often associated with gene repression, whereas DNA hydroxymethylation may facilitate transcription (Spruijt et al., 2013; Mendonca et al., 2014).

## Epigenetics and Sexual Differentiation of the Brain

A transient perinatal exposure to testosterone or its metabolite, estradiol, causes many of the best-studied sex differences in rodent brains, and recent evidence suggests that epigenetic mechanisms underlie many of these hormonal effects (McCarthy et al., 2009; McCarthy and Nugent, 2015; Forger, 2016, 2018). For example, sex differences in the preoptic area of the hypothalamus are disrupted by injecting a DNA methyltransferase inhibitor directly into the brains of newborn rats or mice during the critical period for sexual differentiation (Nugent et al., 2015; Mosley et al., 2017). Similarly, a neonatal disruption of histone acetylation (again, by inhibiting the enzymes that place these marks) prevents the development of sex differences in male rat copulatory behavior (Matsuda et al., 2011), and in size of the bed nucleus of the stria terminalis in mice, a brain region linked to male sexual behavior (Murray et al., 2009). These findings suggest that sexual differentiation of the brain requires orchestrated changes in DNA methylation and histone acetylation.

In another approach, epigenetic marks have been compared between males and females. Based on whole-genome surveys, both histone methylation and DNA methylation patterns differ by sex in the mouse preoptic area (Ghahramani et al., 2014; Shen et al., 2015). Treating newborn female mice with testosterone partially masculinizes the DNA methylation pattern present in adulthood (Ghahramani et al., 2014), and sex differences in the methylation of specific genes also are reversed by neonatal treatment with gonadal steroids in rats (Schwarz et al., 2010). Steroid hormones alter the expression or activity of enzymes that place epigenetic marks (Kolodkin and Auger, 2011; Nugent et al., 2015; Bramble et al., 2016), which may be the mechanism whereby hormones affect the epigenome.

One study in rodents hints at a role for gender in brain epigenetics. Mother rats lick their male neonates more than females (Moore and Morelli, 1979), and the amount of maternal care a rat pup receives affects DNA methylation of the estrogen receptor alpha gene in the brain (Champagne et al., 2006; Kurian et al., 2010). Edelmann and Auger (2011) randomly assigned some newborn females to receive the extra attention normally given to males by simulating maternal licking using a paintbrush. This did, in fact, masculinize the DNA methylation pattern and expression of the estrogen receptor alpha gene in the amygdala of the treated females (Edelmann and Auger, 2011). Being treated differently by your parents based on your perceived sex is an aspect of gender. In this case, however, the differential treatment is based on the odor of the neonate’s urine (Moore, 1985), which in turn is due to differences in circulating testosterone (i.e., sex).

Some sex differences in the brain are independent of gonadal hormones, and are instead due to sex chromosome complement (Arnold et al., 2003; Cisternas et al., 2018). Similarly, sex chromosomes influence the expression of epigenetic enzymes and cause sex differences in the epigenome of rodents and flies (Xu et al., 2008a,b; Jiang et al., 2010; Lemos et al., 2010; Arnold, 2012). Thus, based on animal studies, both major determinants of biological sex (sex chromosomes and gonadal steroids) contribute to differences in the epigenome.

Information on sex differences in the human brain epigenome is very limited. During some stages of human fetal development, the brains of males and females differ in both DNA methylation and hydroxymethylation (Spiers et al., 2015, 2017). Because these differences are seen before birth, and presumably prior to social influences, these are “sex differences.” There are also differences in epigenetic marks in the prefrontal cortex of men and women (Lister et al., 2013; Xu et al., 2014; Gross et al., 2015). Adults have had plenty of gendered experiences, however, so whether these differences are due to sex or gender is not clear. In the next section, we will consider how gender could – and probably does – leave an epigenetic imprint on the brain. We present three specific gendered experiences/exposures occurring at different periods of human development, and for which there are data demonstrating epigenetic effects of those experiences/exposures in animal or human studies.

## Gendered Experiences and Exposures

### Early Life Stress

A growing literature demonstrates that early life stress leaves an epigenetic signature (Roth et al., 2009; Lutz et al., 2018). For example, rodents separated from their mothers throughout early life have reduced DNA methylation and altered gene expression in adulthood within a stress-regulatory brain region (Murgatroyd et al., 2009). Early life maltreatment – being stepped on and ignored by the mother – also alters DNA methylation in genes associated with learning and cell growth, as well as expression levels of epigenetic enzymes in the rat prefrontal cortex (Roth et al., 2009; Blaze and Roth, 2013; Blaze and Roth, 2017).

Similar observations have been made in humans. Compared to children raised by their biological parents, children raised in orphanages have higher DNA methylation of genes associated with immune response, mood, and social behaviors (Naumova et al., 2012). These findings are based on analyses of blood lymphocytes, however, which are often used for this kind of work in humans given the difficulty of obtaining brain samples. In another approach, DNA methylation was compared in the brains of adults who died by suicide, with or without a history of childhood abuse. Those who experienced childhood abuse had decreased hydroxymethylation and expression of the kappa opioid receptor gene in the cortex, suggesting epigenetic programming by a history of early life maltreatment (Lutz et al., 2018).

This work is relevant to the question of whether gender leaves an epigenetic imprint on the brain because the sex of a baby may significantly affect the likelihood that it will face early life stress (Jeffery et al., 1984; van Balen and Inhorn, 2003; Puri et al., 2011). In recent history, for example, China’s “one child policy” resulted in the abandonment of many girls and sharply skewed sex ratios within orphanages (Johnson et al., 1998; Chen et al., 2015). Similarly, during the Great Chinese Famine, families preferred to spend their limited resources on boys, leading to disparities in disability and illiteracy between men and women a generation later (Mu and Zhang, 2011). Treating children differently based on their biological sex is an important part of our definition of gender. Thus, exposure to early life stress changes the neural epigenome, and early life stress can be a gendered experience.

### Environmental Endocrine Disruptors

It is nearly impossible in industrialized societies to avoid exposure to environmental endocrine disruptors such as bisphenol A, phthalates, and parabens. In rodents, developmental exposure to bisphenol A alters DNA methylation in the brain, and changes the expression of DNA methyltransferases in a brain region-specific manner (Yaoi et al., 2008; Kundakovic et al., 2013; Zhou et al., 2013; Walker and Gore, 2017). Moreover, phthalate exposure during adolescence reduces levels of the epigenetic regulatory protein, methyl CpG binding protein 2, and alters social and fear behaviors in rats (Betz et al., 2013). Environmental endocrine disruptors therefore are clearly capable of altering the brain’s epigenome and, to the extent that exposure to these chemicals is gender-based, epigenetic changes may also be gendered.

Interestingly, bisphenol A, phthalates, and parabens are commonly found in cosmetics, scented lotions, nail polish, and feminine care products. There is a vast difference in the use of personal care products between women and men in many parts of the world, and women do, in fact, have higher urinary levels of phthalates and parabens than men (Calafat et al., 2010; Biesterbos et al., 2013; Saravanabhavan et al., 2013). The application of lotions and cosmetics acutely increases levels of urinary paraben concentrations (Meeker et al., 2013), and the difference in urinary levels between males and females emerges in adolescence – the age at which many girls start experimenting with cosmetics and skin care products (Calafat et al., 2010; Dewalque et al., 2014).

The elevated phthalates and parabens in women is likely related to their greater cosmetic use, but is this due to sex or gender? We would say “sex” if, for example, sex chromosomes or gonadal hormones control the desire to use cosmetics, or alter the metabolism or storage of these chemicals in the body. On the other hand, gender is at play if the difference is primarily based on social expectations. Evidence strongly suggests a role for gender because societal norms for cosmetic use vary over time and geography: cosmetics were used by men in ancient Egypt, at the French court in the 17th and 18th centuries, and by British military officers (Carter, 1998; Tapsoba et al., 2010; Ribechini et al., 2011). Very recently, cosmetic use has again become acceptable among men in Western societies (Souiden and Diagne, 2009). Thus, societal gender norms influence cosmetic use. Although no human studies have directly addressed this question, there may well be epigenetic consequences of gendered exposure to cosmetics and other environmental chemicals.

### Alcohol Consumption

Throughout the world, men are more likely to consume alcohol than are women (Wilsnack et al., 2009). A recent meta-analysis found that 39% of men and 25% of women globally are drinkers; moreover, men are more likely to drink excessively, and the increase in disease burden due to alcohol consumption is three times higher in men than in women (GBD 2016 Alcohol Collaborators, 2018). This could reflect sex differences: rodents and non-human primates show sex differences in voluntary alcohol consumption, and gonadal hormones influence preference for an alcohol solution in rodents (Forger and Morin, 1982; Morin and Forger, 1982; Juarez et al., 1993; Ford et al., 2004). Critically, however, the difference in drinking rate between men and women varies enormously by location. In Nepal, for example, men are 14 times as likely as women to be drinkers, whereas in Sweden, the prevalence of drinking is nearly equal between men and women (GBD 2016 Alcohol Collaborators, 2018). Societal factors therefore play a large role, and alcohol consumption can safely be categorized as a gendered behavior in many human societies.

The link to epigenetic changes in the brain in this case is relatively strong. Several studies have reported changes in DNA methylation and histone modifications in the postmortem human brain in association with chronic alcohol consumption (Ponomarev, 2013; Tulisiak et al., 2017). As in most human studies, these are correlations, so it remains possible that alcohol consumption does not *cause* epigenetic changes in the human brain, but that existing epigenetic differences predispose some people to drink. This is where animal studies are again very helpful: many rodent studies in which animals are randomly assigned to ethanol exposure demonstrate a causal relationship between acute or chronic ethanol consumption and epigenetic changes in the brain (Pandey et al., 2008; Kyzar et al., 2016).

## Connecting the Dots

**FIGURE 1:**
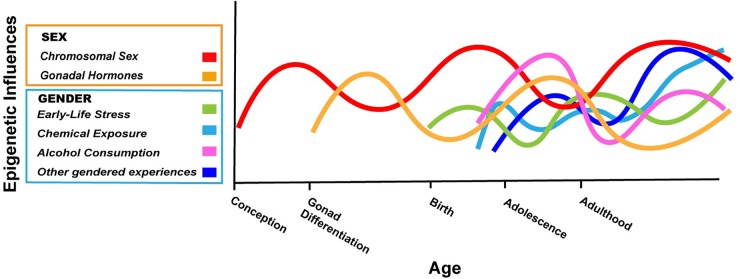
Hypothetical depiction of the complex interplay of sex and gender on the brain epigenome throughout the lifespan. Chromosomal sex is determined at conception and can have effects on the epigenome throughout life (red). The gonads differentiate after the first 10 weeks of fetal life in humans; thereafter, sex differences in gonadal hormones can have acute or lasting effects on the epigenome (gold). The gendered experiences described in this review start as early as birth (early life stress based on gender; green) and continue into adolescence and adulthood (cosmetic use, alcohol consumption; light blue, purple). Many other gendered experiences not explicitly addressed in this review will also impact the neuroepigenome (dark blue). The relative contribution of various factors and how they may change throughout development are not known, but the effects of biological sex and gender will interact in myriad ways throughout life. In some cases, gender may amplify epigenetic differences due to sex, whereas in other cases, gendered experiences may counteract differences in the epigenome based on biological sex. Not shown here is the fact that with our current ability to know the sex of an unborn child, gender can start before birth (Al-Akour, 2008).

The argument we are making is that boys and girls, and men and women, have different exposures and experiences based on societal expectations or perceived expectations (i.e., gender), and that some of these exposures/experiences are known to cause epigenetic changes in the brain based on carefully controlled animal studies. In a few cases, the gendered exposures/experiences have also been associated with epigenetic changes in humans, although most studies are correlational. We have presented just three examples above, but countless experiences/exposures will differ based on gender over a lifetime, and they will interact in complex ways with one another and with the epigenetic consequences of biological sex (Figure 1).

A logical extension of this argument is that variations in gender *within* a sex will also affect the epigenome. For example, cosmetic use among Western women varies from zero to many products a day and correlates with gender expression and sexuality (Loretz et al., 2005; Moore, 2006). If cosmetics cause epigenetic changes, those changes will vary not just between sexes, but also within sex, across cultures, and over the lifespan. Indeed, any differences in the brain between men and women – including those in the epigenome – must be viewed within a social, historical, and developmental context (Springer et al., 2012; Rippon et al., 2014).

Our three examples given above emphasize *exposures* that differ by gender, because these are more likely to have been modeled in animal studies (and therefore to have applicable epigenetic data). However, gender is multi-dimensional, and any aspect (gender roles, identities, beliefs, etc…) may affect the epigenome. Epigenetic modifications are a way for experience to alter gene expression and, taken together, it seems inescapable that gender will leave an epigenetic imprint on the brain.

That said, few studies have directly examined differences in epigenetic marks in the brains of men and women, and none have attempted to separate the contributions of sex and gender. Demonstrating a *causal* relationship between gender and human brain epigenetics will be very challenging, because this will require not only an experimental design, but also brain samples collected at the relevant time point(s). Several authors have proposed methods or best practices for studying effects of gender on biological outcomes, and inroads have been made in separating the effects of sex and gender on disease risk (e.g., Krieger, 2003; Rippon et al., 2014; Pelletier et al., 2015). Given our lifetimes of layered gendered experiences, and their inevitable, iterative interactions with sex, it may never be possible to completely disentangle the effects of sex and gender on the human brain epigenome. We can start, however, by including gender in our thinking any time a difference between the epigenome of men and women is reported.

## Author Contributions

LRC, CC, and NF conceptualized and wrote the manuscript.

## Conflict of Interest Statement

The authors declare that the research was conducted in the absence of any commercial or financial relationships that could be construed as a potential conflict of interest.
